# Diagnostic dilemma in Broad Ligament Leiomyoma with Cystic Degeneration

**DOI:** 10.12669/pjms.302.4361

**Published:** 2014

**Authors:** Shabeen Naz Masood, Yasir Masood, Janta Mathrani

**Affiliations:** 1 Dr. Shabeen Naz Masood, MBBS, MCPS, FICS, FCPS, PhD, DCPS, Consultant Obstetrician & Gynecologist, Medical Superintendent,Sobhraj Maternity Hospital, KMC, Karachi, Pakistan.; 2 Dr. Yasir Masood, MBBS, Ziauddin Medical University, Karachi, Pakistan.; 3 Dr. Janta Mathrani, MBBS, MCPS trainee, Department of Obstetrics & Gynecology, Sobhraj Maternity Hospital, KMC, Karachi, Pakistan.

**Keywords:** Benign Uterine Tumor, Cystic Degeneration, Leiomyoma, Ovarian Tumor

## Abstract

Fibroids are smooth muscle benign tumors; most commonly arise from uterus but may also rise from extra uterine sites like broad ligament. This case report of broad ligament myoma with extensive cystic degeneration is presented for its rarity and diagnostic challenges as they mimic pelvic adenexal tumors. Mrs. X, 43 years old p_5+2_, asymptomatic women with no co-morbids presented with mass in abdomen. The MRI showed mix attenuation mass of 19.7 x 16.8 x 13.7cms arising from right side of uterus extending up to epigastrium, with cystic and solid components and ascitic fluid. Resection of mass with abdominal hysterectomy with bilateral salphingo oophrectomy was done. No local or abdominal organ metastases were seen. Histopathology showed left broad ligament leiomyoma weighing 4000 grams with cystic degeneration.

***Conclusion:*** Huge broad ligament leiomyoma with cystic degeneration and abdominal ascites may cause diagnostic dilemma with ovarian malignancy. This differential diagnosis must be considered before surgery.

## INTRODUCTION

**Fig.1:**
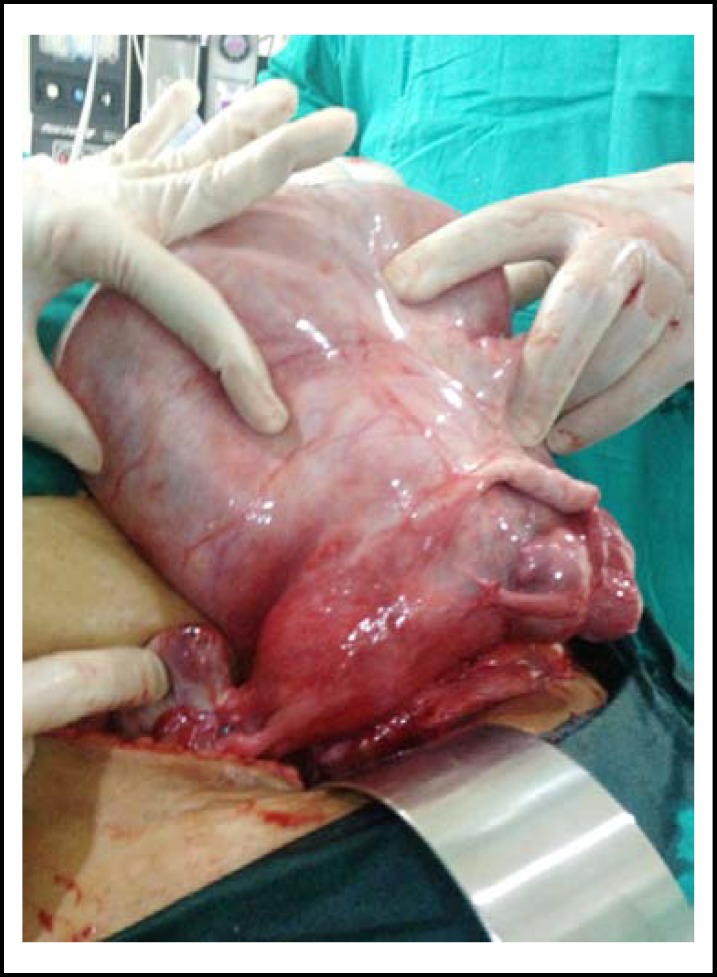
Huge mass with uterus and bilateral ovaries

Leiomyomas are commonly found in 20 – 30% of uterine tumors with typical sonological appearance.^1^ The diagnostic dilemma arises when leiomyomas undergo degenerative changes. This is a case report of huge leiomyoma with massive cystic degeneration resulting in diagnostic confusion with ovarian malignancy on clinical evaluation and radiological findings. Fig.1

## HISTORY

Mrs. X para 5+2, asymptomatic, hypertensive obese woman with previous cesarean deliveries and tubal ligation (09 years back), presented with ultrasound (U/S) showing an ovarian tumor of huge dimensions. She was regularly menstruating, had no urinary, bowel, sexual or weight related complaints. She got the ultrasound done as her abdomen was increasing in size. She had consulted many gynecologists to avoid surgery. Abdominal examination revealed a hard mass extending from pelvis up to xiphisternum and laterally extending into the flanks, shifting dullness was not present. On pelvic examination the uterus was difficult to identify separately from the mass; it was non-tender, fixed and all the fornices were full with hard solid palpable nodules. There was no palpable lymphadenopathy.

The CA 125, Carcino Embryonic Antigen (CEA), serum alpha foeto protein HE_4 _levels were normal. ultrasound on 14-06-2013 reported, a large cystic cum solid multiseptate mass extending from right side of uterus up to the right hypochondrium of about 19.7 x 16.8 x 13.7cm. Color Doppler showed low resistant vascularity in thin parts. R.I was 0.43.

High resistant vascularity was found in left ovary measuring 5.8 x 4.0 cm, free fluid in cul-de-sac and para colic gutters, with no abdominal visceral involvement. MRI on 19-06-2013 showed a well defined left adenexal mass of mixed attenuation predominantly cystic, extending up to the level mid pole of the kidney anteriorly. It measured 14.0 x 20.0 x 20.0 cm in anteroposterior, transverse and craniocaudal dimensions. After anesthesia fitness the surgery was planned. On laprotomy a transverse rectus cutting abdominal incision was given. About 500 ml straw colored fluid was aspirated from abdominal cavity.

On opening the peritoneum the appearance of the tumor was as if the guts were adherent to the anterior surface of the tumor. The mass was arising from the pelvis and extending up to xiphisternum with lateral extensions to both the para colic gutters. It had solid cum cystic consistency and multiple cysts were filled with straw colored fluid. Normal sized uterus was attached to the lower right lateral pole of the mass with a normal looking right and left ovary. Left lateral extension of the mass was encroaching into the retroperitoneum, the pedicle of the tumor was arising from the left lateral uterine border extending into broad ligament.

The mass was removed and subtotal hysterectomy was done due to adherence of bladder with the lower uterine segment. Surrounding structures like anterior surface of bladder, intestines, liver and diaphragm were normal looking. Para aortic lymph nodes were not palpable. After securing homeostasis, abdomen was closed in layers.

***Histopathology Report:*** **A large** cyst about 19.5 x 16.5 x 6cm in dimension; weighing 4000grams with solid areas measuring 9.5 x 9.3cm was reported. The cut surface was grey white with whorled appearance. This multilocular cyst contained yellow serous fluid. Secretory endometrium, unremarkable ovaries and fallopian tubes. No evidence of malignancy. Cytology of fluid showed no malignant cells. Immunohistochemical markers showed Caldesmon - Positive in spindle cells.

## DISCUSSION

Uterine leiomyoma can undergo cystic degeneration and is said to be found in 4% of all types of degenerations.^2^ The commonest type of degeneration is hyaline seen in 60% of patients.^3^

Usually uterine leiomyoma does not present a clinical and sonological diagnostic challenge. However, when fibroids undergo massive cystic degeneration they may present clinical and sonologic diagnostic difficulties. In this case report the asymptomatic presentation, with a huge palpable mass and ultrasound appearance of extensive tumor with solid cum cystic areas, presence of peritoneal free fluid mimicking Pseudo-Meig's syndrome, raised the suspicious of ovarian malignancy.

The patient’s normal readings of CA 125, CEA and alpha fetoprotein levels; being non-specific tumor markers could not be banked upon. Serum HE_4_ levels however were <70 pmol/L.

MRI is a useful diagnostic tool; Leiomyomas with cystic degeneration typically show decreased T1W and high signal intensity on T2-weighted images, and the cystic areas do not show enhancement on contrast image.^3^^,^^4^

**Fig.2:**
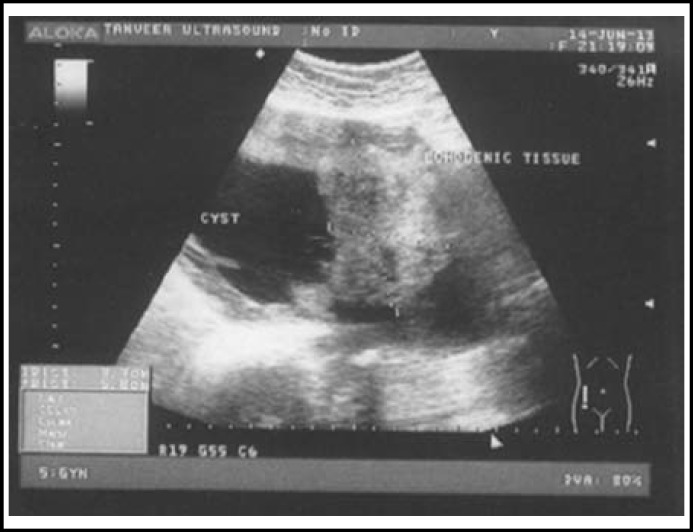
Ultrasound showing a large solid cum cystic mass of 19.7x16.8x13.7 cm with multiple thin septae inside the cyst

Intraoperatively the irregular shape, solid cum cystic appearance and the left lateral extension of the mass caused difficulty in distinguishing from retroperitoneal malignant encroachment of tumor. Preoperative identification of feeding or draining vessels arising from the myometrium could be helpful in distinguishing an intraligamentary leiomyoma from a retroperitoneal tumor.^5^ Knowledge of unusual presentation of the benign degenerated fibroids is essential to the clinicians; to help differentiate benign from malignant tumor entities.^6^ Intraoperative finding of ascites with the huge tumor mimicked ovarian malignancy. However, the attachment of the tumor to the lateral border of uterus along with bridging vessel sign (vessels bridging the mass and myometrial tissue seen in Fig.1) can provide a clue to the diagnosis of degenerated broad ligament fibroid. Intraoperative high index of suspicion and careful search for the above mentioned signs can salvage ovaries and uterus in younger patients.

## CONCLUSION

Huge broad ligament leiomyoma with cystic degeneration may present diagnostic difficulties in differentiating ovarian malignancies even on ultrasound and MRI. This differential diagnosis must be considered prior to surgical management.

## References

[1] Szklaruk J, Tamm EP, Choi H, Varavithya V (2003). MR imaging of common and uncommon large pelvic masses. Radio Graphics.

[2] Ueda H, Togashi K, Konishi I, Kataoka ML, Koyama T, Fujiwara T (1999). Unusual appearances of uterine leiomyomas: MR imaging findings and their histopathologic backgrounds. Radiographics.

[3] Okizuka H, Sugimura K, Takemori M, Obayashi C, Kitao M, Ishida T (1993). MR detection of degenerating uterine leiomyomas. J Comput Assist Tomogr.

[4] Wilde S, Scott-barrett S (2009). Radiological appearances of uterine fibroids. Indian J Radiol Imaging.

[5] Torashima M, Yamashita Y, Matsuno Y (1998). The value of detection of flow voids between the uterus and the leiomyoma with MRI. JMRI.

[6] Fasih N, Prasad Shanbhogue AK, Macdonald DB, Fraser-Hill MA, Papadatos D, Kielar AZ (2008). Leiomyomas beyond the uterus: unusual locations, rare manifestations. Radiographics.

